# Acuity Differences Among Newly Admitted Medicare Residents in Rural and Urban Skilled Nursing Facilities

**DOI:** 10.1093/geroni/igab046.1592

**Published:** 2021-12-17

**Authors:** Yvonne Catharina Jonk, Andrew Coburn, Catherine McGuire, Deborah Thayer, Karen Mauney

**Affiliations:** 1 University of Southern Maine, Muskie School, Portland, Maine, United States; 2 University of Southern Maine, Portland, Maine, United States

## Abstract

Using the 2015 national Minimum Data Set Version 3.0, the Area Health Resources Files, the 2015 Provider of Services File, and the Rural-Urban Commuting Area codes, this study assessed rural-urban differences in newly admitted, Medicare skilled nursing facility (SNF) residents’ functional status, cognitive performance, and behavioral issues using self-performance, early loss, and late loss Activities of Daily Living (ADLs); the Cognitive Function Scale (CFS); and indicators of aggression, psychosis, or wandering, respectively. The study evaluated 686,881 unique patient assessments for newly admitted Medicare SNF residents across 15,157 facilities in 47 states. Negative binomial and generalized linear models with state fixed effects and clustering by SNFs were used to evaluate rural-urban acuity differences before and after adjusting for socio-economic factors; admission source, and market area characteristics. Compared to urban SNF residents, rural residents were more likely to be cognitively impaired (45% Isolated Small Rural, 44.5% Small Rural, 41% Large Rural, 38.8% Urban), and have behavioral issues (6.7% rural, 4.8% urban). Unadjusted and adjusted regression models confirmed bivariate findings that rural SNF residents were less functionally impaired (IRR range: 0.974-.987), but had more cognitive and behavioral issues in more remote rural locations than urban. The (unadjusted) odds of cognitive impairment were 1.1-1.3 times higher for residents of rural vs urban SNFs; while the odds of having any one of the behavioral issues were 1.2-1.6 times higher in more remote rural locations. The capacity of rural SNFs to manage complex cognitive and behavioral problems deserves further research.

